# Pre-treatment inflammatory markers can identify the risk of immune-related toxicity in non-small cell lung cancer patients receiving immune checkpoint inhibitor therapy

**DOI:** 10.3389/fonc.2026.1867253

**Published:** 2026-07-29

**Authors:** XueYuan Zhang, Jia Song

**Affiliations:** Cancer Center, The Fifth Hospital Affiliated to Wenzhou Medical University, Lishui Municipal Central Hospital, Lishui, Zhejiang, China

**Keywords:** immune checkpoint inhibitors, immune-related adverse events, inflammatory markers, nomogram, non-small cell lung cancer

## Abstract

**Objective:**

This work intended to explore how peripheral blood inflammatory markers at baseline predict the occurrence of immune-related adverse events (irAEs) in patients with non-small cell lung cancer (NSCLC) treated with immune checkpoint inhibitors (ICIs).

**Methods:**

This retrospective study included 285 patients with stage III–IV NSCLC who received ICI therapy at our hospital from January 2020 to December 2023. Based on the presence of irAEs, patients were assigned to an irAEs group (91 patients) or a non-irAEs group (194 patients). Baseline clinical data and peripheral blood inflammatory markers (including CRP, TNF-α, IL−2, IL−4, IL−6, NLR, MLR, PLR, and SII) were collected within one week before treatment initiation. LASSO regression was applied for variable selection, followed by multivariate logistic regression to identify independent predictors of irAEs. A nomogram prediction model was constructed and its discrimination, calibration, and clinical utility were assessed using AUC, calibration curves, and decision curve analysis, with internal validation via bootstrap resampling.

**Results:**

91 patients (31.93%) developed irAEs, with the most common being pneumonia (21 cases). OS and PFS did not significantly differ between the irAEs and non-irAEs groups, according to survival analysis (*P*>0.05). CRP, IL-4, NLR, IL-2, and TNF-α were found to be independent predictors of irAEs (*P* < 0.05) using multivariate logistic regression. The AUC of the nomogram was 0.896 (95% CI: 0.859–0.934). The bootstrap-corrected AUC was 0.891 (95% CI: 0.851–0.930). The decision curve showed a net benefit greater than 0 across the entire threshold range of 0.00–0.08.

**Conclusion:**

The nomogram model, based on pre-treatment peripheral blood inflammatory markers (CRP, IL-4, NLR, IL-2, and TNF-α), demonstrated good discriminatory and calibration capabilities in the validation cohort. However, this model should be regarded as an exploratory tool designed to generate hypotheses for identifying patients at risk of developing immune-related adverse events. External validation is required before any clinical application.

## Introduction

Lung cancer, which accounts for around 12% of new cancer cases and roughly 19% of cancer deaths worldwide, continues to be one of the primary causes of mortality associated with malignant tumors. It places a heavy burden on the public health system (1).Between 85% and 90% of instances of lung cancer are non-small cell lung cancer (NSCLC). The majority of patients are detected at an advanced stage and miss out on the chance for drastic surgical treatment because of its subtle onset and unusual early symptoms (2). Immunotherapy based on immune checkpoint inhibitors has made significant strides in the treatment of advanced lung cancer in recent years. Immune checkpoint inhibitors, whether used alone or in conjunction with chemotherapy, have greatly increased the objective response rate and overall patient survival in both first-line and second-line treatment. As a result, they are now a crucial part of the standard treatment plan for advanced non-small cell lung cancer (3).

Immune checkpoint inhibitors, however, may potentially cause abnormally activated immune responses that target healthy tissues, resulting in immune-related adverse events (irAEs), even if they stimulate the body’s immune system to destroy tumor cells (4). Numerous organ systems, including the skin, gastrointestinal tract, liver, lungs, endocrine glands, cardiovascular system, and neurological system, may be impacted by these toxic reactions. The occurrence time and severity of these events are highly uncertain. Mild cases may present as manageable rashes or diarrhea, while severe cases may develop into immune-related pneumonia, myocarditis, or pituitary inflammation, and even be life-threatening (5). irAEs not only affect patients’ treatment compliance and quality of life but also may lead to the interruption or termination of immune checkpoint inhibitor treatment, weaken anti-tumor efficacy, and limit the maximization of the clinical benefits of immunotherapy (6). Therefore, how to effectively identify high-risk populations for irAEs before treatment has become an important issue in clinical practice of immunotherapy.

Currently, the monitoring of irAEs in clinical practice mainly relies on the identification of symptoms during the treatment process and imaging and laboratory tests, lacking reliable indicators that can effectively predict the risk of their occurrence before the treatment (7). Current biomarkers including PD-L1 expression and tumor mutational burden are correlated with treatment efficacy, yet their relationship with treatment-related toxicity remains understudied. Moreover, due to factors such as detection costs and the difficulty in obtaining tumor tissues, these biomarkers are difficult to be widely applied (8). The immunotherapy process is significantly impacted by the systemic inflammatory state, according to studies (9). Systemic inflammation not only affects the tumor microenvironment and immune response effects, but may also be related to the susceptibility to immune-related toxicities (10). The systemic immune inflammation index (SII), the ratio of neutrophils to lymphocytes (NLR), the ratio of platelets to lymphocytes (PLR), and the ratio of peripheral blood mononuclear cells to lymphocytes (MLR) are examples of inflammatory indicators that can easily and non-invasively reflect the state of the body’s immune and inflammatory balance. They show good potential in predicting the prognosis and efficacy of various solid tumors. However, research on their capacity to predict the risk of irAEs is scarce (11).

In light of this, this investigation begins with systemic inflammatory markers prior to treatment. It investigates the relationship between inflammatory-related indicators like NLR, PLR, and SII and the incidence of irAEs, evaluates their predictive efficacy, and further integrates clinical pathological features to build a risk prediction model through an observational analysis of the clinical data of patients with non-small cell lung cancer who received immune checkpoint inhibitor treatment. The aim is to help clinicians identify high-risk populations for irAEs early and improve the comprehensive benefits and safety of immunotherapy for advanced lung cancer.

## Materials and methods

### General information

**Figure 1:**
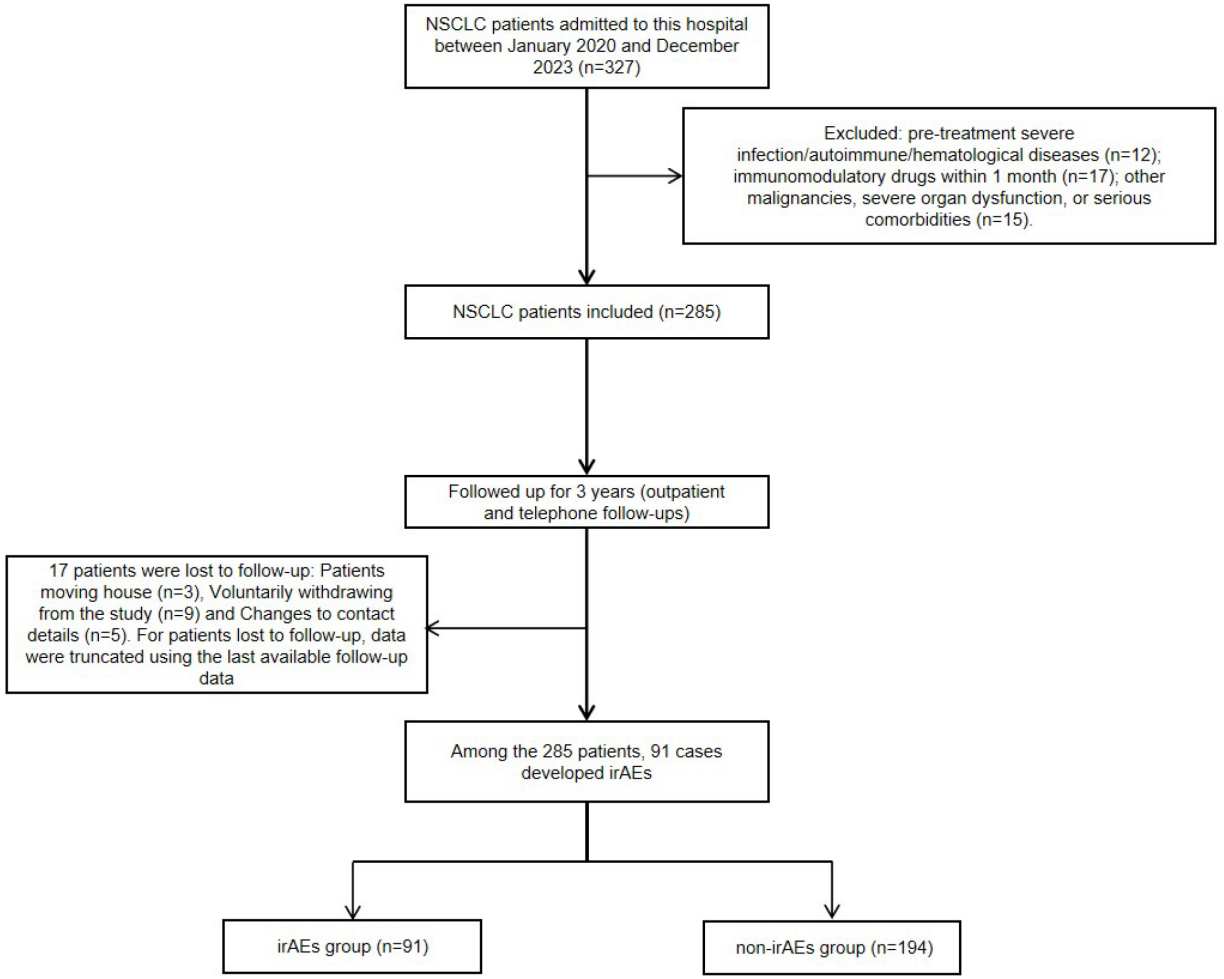
Patient screening flowchart.

This study enrolled 327 NSCLC patients hospitalized in our center from January 2020 to December 2023. After excluding 42 individuals who failed to meet the inclusion criteria, 285 participants were enrolled. For the 17 patients with missing survival data, the analysis was based on the last available follow-up record. The follow-up status for these patients was set to the last known value at the time of the last contact. All eligible patients were finally allocated into the irAEs group (n=91) and non-irAEs group (n=194) depending on whether irAEs occurred (**Figure 1**). In line with the criteria for multivariate Logistic regression analysis, a minimum of 10 outcome events per independent variable (EPV≥10) is required to guarantee the stability of the prediction model. This study expected to include no more than 8 independent variables for analysis. Relevant prior research has indicated that the incidence rate of irAEs in patients is roughly 30%–40% (12), with EPV = 91/8≈11. At least 267cases were needed, considering the actual situation of our hospital, and finally 285 cases were included. This study has been approved by the medical ethics committee of our hospital (Ethics Number: 2026(I)-134-01).

Inclusion criteria: 1) Aged 18 years or older; 2) Histopathologically confirmed diagnosis of NSCLC, and with TNM stage III-IV (13); 3) First-time receiving immune checkpoint inhibitor treatment, with a clear treatment plan; 4) Completed peripheral blood related index tests within 1 week before treatment.

Exclusion criteria: 1) Had severe infection, or hematological diseases before treatment; 2) Had used glucocorticoids, immunosuppressants, or other drugs affecting the body’s immune function within 1 month before treatment; 3) Had other primary malignant tumors, severe liver and kidney dysfunction, cardiovascular and cerebrovascular emergencies, or other serious underlying diseases.

### Treatment plan

Treatment decisions were based on clinical guidelines and patient-specific factors at the time of initiation. Patients with a programmed death ligand 1 (PD-L1) tumor proportion score (TPS) of 50% or higher received pembrolizumab monotherapy (200 mg every three weeks) as first-line treatment. For patients with PD-L1 TPS <50% or those who progressed after first-line therapy, alternative regimens included: pembrolizumab (2 mg/kg every three weeks), nivolumab (3 mg/kg every two weeks), or atezolizumab (1,200 mg every three weeks), administered alone or in combination with chemotherapy at the physician’s discretion. Treatment continued for up to 2 years until imaging or clinical disease progression, occurrence of intolerable toxicity, or death from any cause.

### Data collection

Data of the patients were collected, including the following: (1) Baseline data, Including age, gender, Karnofsky Performance Status (KPS) score, TNM stage, pathological type, history of diabetes, history of hypertension, chronic obstructive pulmonary disease (COPD), smoking history, drinking history, type of immune checkpoint inhibitors, targeted therapy. (2) Pathological and imaging parameters: The proportion of tumor cells in the division stage in tumor tissue (Ki-67 index). (3) Laboratory data: Collect the results of peripheral blood tests for all patients within one week prior to the immunotherapy session, including white blood cell count (WBC), neutrophil count (NEU), monocyte count (MONO), lymphocyte count (LYM), platelet count (PLT), lactate dehydrogenase (LDH), albumin (ALB), and C-reactive protein (CRP), tumor necrosis factor-α (TNF-α), interleukin-2 (IL-2), interleukin-4 (IL-4), the ratio of neutrophils to lymphocytes (NLR), the ratio of monocytes to lymphocytes (MLR), and the ratio of platelets to lymphocytes (PLR); the formula for calculating the systemic immune inflammation index (SII) is (neutrophil count × platelet count)/lymphocyte count.

### Follow-up methods and criteria for irAEs determination

Patients were evaluated in person at three-week intervals and contacted by telephone weekly throughout the follow-up period. All potential adverse events were prospectively recorded in a standardized case report form by the treating physicians at each visit. irAEs ascertainment followed a structured two-step adjudication process. First, the treating physician documented all adverse events with onset date, clinical manifestations, severity grading, and management details. Second, all potential irAEs cases were independently reviewed and confirmed by a multidisciplinary adjudication committee comprising an oncologist, a pulmonologist, and a gastroenterologist, all of whom were blinded to the patients’ baseline inflammatory biomarker levels. Discrepancies were resolved by consensus discussion. This adjudication process was established prior to study commencement to minimize outcome misclassification bias.

irAEs were defined in accordance with the “Management Guidelines for Immune Checkpoint Inhibitor-related Adverse Events” promulgated by the American Society of Clinical Oncology (14), encompassing adverse events associated with ICI drugs with a potential immunological basis and requiring frequent monitoring or potential intervention. The severity of irAEs was graded according to the Common Terminology Criteria for Adverse Events (CTCAE) version 5.0 developed by the Cancer Therapy Evaluation Program of the National Cancer Institute (15) with grades ranging from 1 (mild) to 5 (death). Specific irAEs categories included: pneumonia (new pulmonary infiltrates on imaging with clinical or microbiological evidence of infection exclusion); diarrhea (increase in stool frequency ≥4 times/day or change in stool consistency with infectious etiology excluded); elevated lipase/amylase (lipase >3× upper limit of normal or amylase >2× upper limit of normal without pancreatic duct obstruction); elevated transaminases (ALT or AST >3× upper limit of normal with viral hepatitis excluded); cutaneous reactions (rash, pruritus, or vitiligo requiring topical or systemic intervention); arthralgia/myalgia (musculoskeletal symptoms ≥grade 2 with alternative etiologies excluded); and endocrine-related events (hypothyroidism, hyperthyroidism, hypophysitis, or adrenal insufficiency with hormonal confirmation). The “other events” category comprised immune-related pericarditis (n=1) and immune-related pancreatitis (n=1), both confirmed by imaging and biopsy, respectively.

### Study endpoints

This study primarily assessed irAEs and examined how peripheral blood markers relate to the risk of developing these events. Peripheral blood indicators and the incidence of irAEs are assessed, with irAEs serving as the major assessment. Overall survival (OS) is the secondary endpoint. Progression-free survival (PFS) is one of the additional endpoints. The Response Evaluation Criteria in Solid Tumours (RECIST) version 1.1, which is divided into four categories—complete response (CR), partial response (PR), stable disease (SD), and disease progression (PD)—will be used to evaluate efficacy. The total of the complete and partial responses (CR + PR) will be used to get the objective response rate (ORR). Three years was chosen as the study’s follow-up period. OS was defined as the duration between the beginning of treatment and death, whereas PFS was defined as the duration between the beginning of treatment and either disease progression or death from any cause.

### Statistical analysis

R version 4.5.2 was used for all data analyses. After testing for normality and variance homogeneity, normally distributed continuous variables with equal variances were presented as mean ± SD and compared between groups using the independent samples t-test, whereas those deviating from normality were expressed as median (IQR) and compared using the Mann−Whitney U test. Categorical variables were described using counts and percentages, with intergroup comparisons performed via chi−square or Fisher’s exact tests, as appropriate. Among the 285 patients included in the analysis, the missing rate for each variable was all below 5%. For the small number of missing values in the continuous variables, the multiple imputation method (Multiple Imputation by Chained Equations, MICE) was used for processing. Based on the predictive mean matching algorithm, 5 complete data sets were generated, and the final results were obtained by taking the combined estimates; for the missing values of categorical variables, the mode was used to fill in.

Further Lasso regression was performed on the variables to reduce dimensionality and eliminate multicollinearity, followed by binary logistic regression to identify independent risk factors for stroke recurrence. A column plot prediction model was constructed based on the regression results. A multivariate logistic regression model was built to determine independent risk factors for irAEs occurrence, and a nomogram prediction model was subsequently established to facilitate personalized prediction of irAEs risk. The model’s discriminative ability was quantified by the area under the receiver operating characteristic curve (AUC) with 95% confidence intervals. Internal validation was performed using 1,000 bootstrap resamples. Model calibration was assessed by comparing predicted probabilities with observed outcomes, and clinical utility was evaluated using decision curve analysis to estimate net benefit across different risk thresholds. *Post-hoc* interpretability analysis was conducted using Shapley additive explanations (SHAP). Survival analysis was conducted using the landmark method. Statistical significance was set at *P* < 0.05.

## Results

### Treatment status

The median follow-up period was 7.81 months (1.0~35.5 months), and it terminated in December 2025. As the disease progressed, 64.21% (183/285) of the patients stopped using ICIs, with a median dose cycle of 4 (1~37) cycles. 89 cases had PR, 119 cases had SD, 74 cases had PD, and one patient had CR. 31.58% was the ORR.

### Incidence of irAEs

91 of the 285 patients with NSCLC had irAEs. A total of seven different types of irAEs were experienced by these 91 patients (**Table 1**): pneumonia (21 cases), diarrhea (17 cases), elevated lipase/amylase levels (16 cases), elevated transaminases (3 cases), cutaneous reactions (17 cases), arthralgia/myalgia (9 cases), endocrine-related events (6 cases) and other events (2 cases). The “other events” category comprised immune-related pericarditis (1 case) and immune-related pancreatitis (1 case).

**Table 1:** Incidence of irAEs.

irAE	*n*	irAEs grading, n (%)					ICIs suspend, n (%)	stop ICIs, n (%)	Systemic hormone/hormone replacement therapy is required, n (%)
		Level 1	Level 2	Level 3	Level 4	Level 5			
Pneumonia	21	5(23.8)	6(28.6)	5(23.8)	2(9.5)	3(14.3)	21(100.0)	15(71.4)	20(95.2)
Diarrhea	17	3(17.6)	8(47.1)	6(35.3)	0	0	14(82.4)	9(52.9)	16(94.1)
Elevated lipase/amylase levels	16	3(18.8)	5(31.3)	6(37.5)	2(12.5)	0	16(100.0)	5(31.3)	13(81.3)
Elevated transaminases	3	0	1(33.3)	2(66.7)	0	0	3(100.0)	2(66.7)	3(100.0)
Cutaneous reaction	17	8 (47.1)	9(52.9)	0	0	0	3(17.6)	0	6(35.3)
Arthralgia/myalgia	9	4(44.4)	3(33.3)	2(22.2)	0	0	4(44.4)	1(11.1)	4(44.4)
Endocrine-related events	6	4(66.7)	2(33.3)	0	0	0	2(33.3)	2(33.3)	5(83.3)
Others	2	0	0	0	1(50.0)	1(50.0)	2(100)	1(50.00)	2(100)

### Comparison of clinical characteristics between the irAE group and the non-irAE group

In the comparison of clinical characteristics between the irAE group and the non-irAE group, the indicators with statistically significant differences (*P* < 0.05) included: age, gender, COPD, type of immune checkpoint inhibitors, combined chemotherapy, CRP, TNF-α, IL-2, IL-4, IL-6, NEU, LYM, MLR, NLR, PL and SII (**Table 2**).

**Table 2:** Comparison of clinical characteristics between patients in the irAE group and non-irAE group.

Variable	irAE group(*n* = 91)	non-irAE group(*n* = 194)	*t/χ^2^*	*P*
Age,Year	68.19 ± 7.65	63.48 ± 8.74	-4.624	<0.001
Gender, n (%)			14.326	<0.001
Male	73 (80.22%)	111 (57.22%)		
Female	18 (19.78%)	83 (42.78%)		
KPS grade	77.24 ± 12.61	77.81 ± 8.59	0.434	0.665
TNM staging, n (%)			0.071	0.790
II~III	31(34.07%)	62(31.96%)		
IV	60(65.93%)	132(68.04%)		
Pathological type, n (%)			0.846	0.358
Squamous cell carcinoma	48(52.75%)	91(46.91%)		
Adenocarcinoma	43(47.25%)	103(53.09%)		
Diabetes, n (%)			1.005	0.316
Yes	12(13.19%)	18(9.28%)		
No	79(86.81%)	176(90.72%)		
Hypertension, n (%)			0.543	0.461
Yes	36(39.56%)	68(35.05%)		
No	55(60.44%)	126(64.95%)		
COPD, n(%)			12.166	<0.001
Yes	28(30.77%)	26(13.40%)		
No	63(69.23%)	167(86.08%)		
History of autoimmune diseases, n (%)			0.934	0.968
Rheumatoid Arthritis	7(7.69%)	4(2.06%)		
Psoriasis/Psoriatic Arthritis	5(5.49%)	2(1.03%)		
Autoimmune Thyroiditis (Hashimoto's)	3(3.30%)	2(1.03%)		
Inflammatory Bowel Disease	2(2.20%)	1(0.52%)		
Sjögren's Syndrome	1(1.10%)	1(0.52%)		
Systemic Lupus Erythematosus	1(1.10%)	0(0.00%)		
Smoking history n (%)			1.787	0.183
Yes	49(53.84%)	88(45.36%)		
No	42(46.15%)	106(54.64%)		
Alcohol Consumption history, n (%)			2.352	0.125
Yes	33(36.26%)	53(27.32%)		
No	58(63.74%)	141(72.68%)		
Type of immune checkpoint inhibitors, n (%)			11.654	<0.001
PD-1 inhibitors	88(96.70%)	159(81.96%)		
PD-L1 inhibitors	3(3.30%)	35(18.04%)		
Targeted therapy n (%)			0.028	0.867
Yes	46(50.55%)	97(50.00%)		
No	45(49.45%)	97(50.00%)		
Combined chemotherapy, n (%)			12.816	<0.001
Yes	70(76.92%)	104(53.61%)		
No	21(23.08%)	90(46.39%)		
WBC, ×10^9^/L	7.39 ± 1.57	7.52 ± 1.63	0.635	0.526
Ki-67 index(%)	40.71 ± 13.65	41.08 ± 13.18	0.218	0.827
LDH, U/L	180.00 ± 39.51	184.56 ± 35.92	0.934	0.352
ALB, g/L	39.84 ± 4.56	40.12 ± 4.80	0.466	0.641
CRP, g/L	6.36 ± 1.07	4.53 ± 1.09	13.385	<0.001
TNF-α, pg/mL	12.40 ± 1.49	10.47 ± 1.33	10.525	<0.001
IL-2, pg/mL	1.64 ± 0.33	1.55 ± 0.29	2.335	0.028
IL-4, pg/mL	2.39 ± 0.43	1.94 ± 0.43	-8.283	<0.001
IL-6, pg/mL	8.19 ± 2.39	6.48 ± 2.14	-5.824	<0.001
NEU, ×10^9^/L	5.02 ± 0.88	4.07 ± 0.69	-9.044	<0.001
MONO, ×10^9^/L	0.57 ± 0.05	0.56 ± 0.042	-1.058	0.292
PLT, ×10^9^/L	248.20 ± 48.57	236.43 ± 55.65	-1.819	0.070
LYM, ×10^9^/L	1.38 ± 0.27	1.68 ± 0.33	8.209	<0.001
MLR	0.43 ± 0.10	0.35 ± 0.06	7.273	<0.001
NLR	3.79 ± 0.99	2.51 ± 0.63	11.274	<0.001
PLR	185.74 ± 47.20	145.20 ± 42.18	6.988	<0.001
SII	928.81 ± 267.31	592.98 ± 208.88	10.567	<0.001

### Survival analysis of the irAE group and the non-irAE group

**Figure 2:**
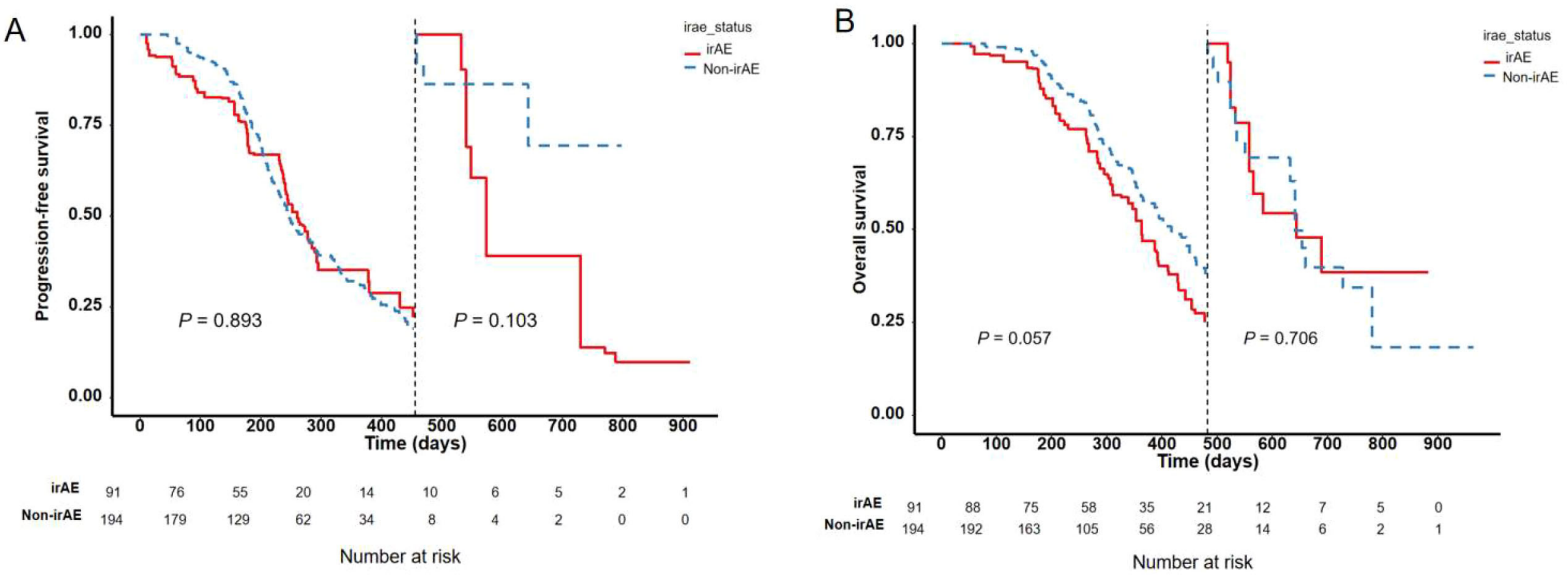
Landmark segmented survival curve. **(A)** Progression-free survival. **(B)** Overall survival.

Over follow-up, 212 tumor progression events occurred (incidence 74.39%): 68 in the irAE group (74.72%) and 144 in the non-irAE group (74.23%), with no significant difference between groups (*P*>0.05). The landmark survival analysis results showed that when evaluating PFS using a 450-day cut-off time, there was no significant difference between the two groups before the cut-off point (*P* = 0.893), and after the cut-off point, there was also no significant difference in PFS in the irAE group (*P* = 0.103) (**Figure 2A**). A total of 111 deaths were recorded, giving an OS rate of 61.05%. The survival rate was 63.73% (58 survivors) in the irAE group and 59.79% (116 survivors) in the non-irAE group (*P*>0.05). When evaluating OS using a 500-day cut-off time, there was no significant difference between the two groups before the cut-off point (*P* = 0.057), and after the cut-off point, the difference was also not statistically significant (*P* = 0.706) (**Figure 2B**).

### Lasso regression analysis for variable selection

**Figure 3:**
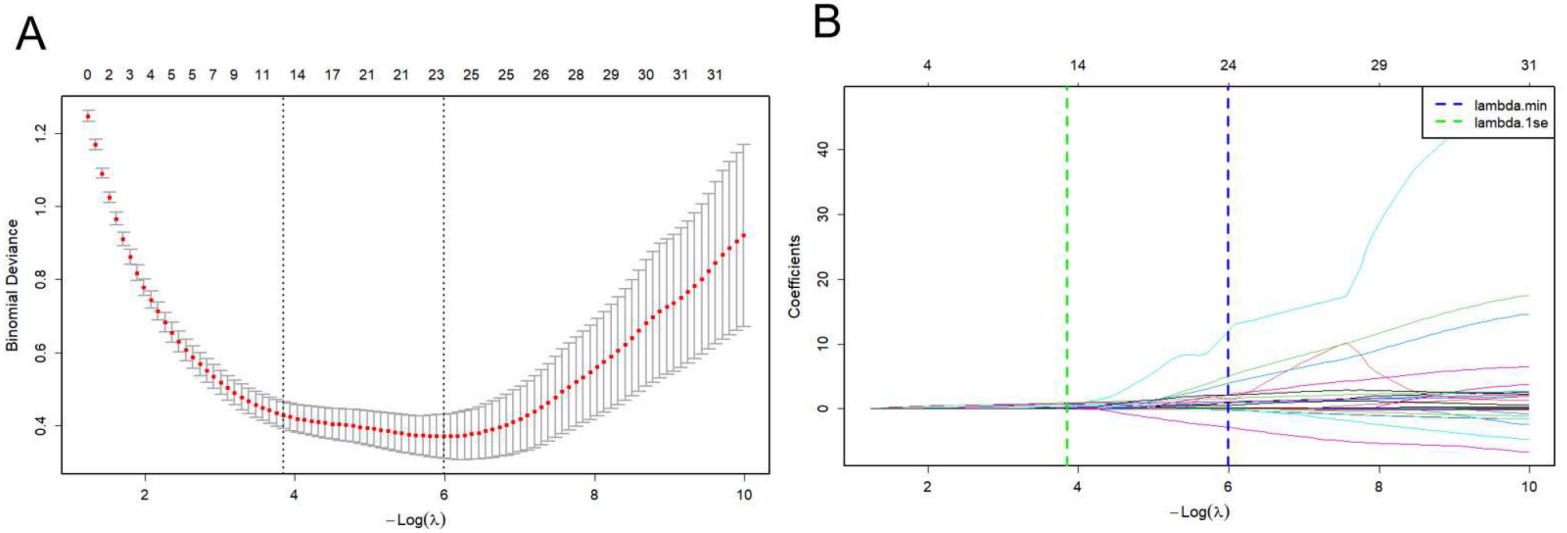
Lasso regression screening. **(A)** Lasso graph. **(B)** Plot of the Lasso regression coefficient profiles.

In LASSO regression, variable selection is conducted on the original dataset, and the final model is then applied to each bootstrap sample. Initial univariate analysis identified 18 indicators with significant intergroup differences (*P* < 0.05). Using cross-validation to minimize the mean square error, the optimal Lasso regression parameter λ was determined as 0.02134567. At this λ value, 8 variables with non-zero coefficients were ultimately retained, including CRP, IL-4, NLR, IL-2, TNF-α, MLR, combined chemotherapy, and COPD (**Figure 3**).

### Collinearity test analysis

The variance inflation factor (VIF) and tolerance values were used to evaluate the 8 significant indicators selected by lasso. A VIF value greater than 5 or a tolerance value less than 0.2 indicates the presence of multicollinearity. All independent variables satisfied VIF < 5 and tolerance > 0.2, indicating that multicollinearity was not present (**Table 3**).

**Table 3:** Test for collinearity.

Characteristics	VIF	Tolerance
CRP	1.282	0.780
IL-4	1.221	0.819
NLR	2.572	0.389
IL-2	1.031	0.970
TNF-α	1.415	0.707
MLR	2.215	0.452
Combined chemotherapy	1.208	0.828
COPD	1.238	0.808

### Multivariate logistic regression analysis

The variables chosen using lasso regression were further examined using multivariate analysis, with the dependent variable being if irAE occurred (with values: no=0, yes=1). The results showed that CRP (OR = 4.93, 95% CI: 2.975~9.184, *P* < 0.001), IL-4 (OR = 5.60, 95% CI: 1.757–19.908, *P* = 0.005), NLR (OR = 5.55, 95% CI: 2.36~14.629, *P* < 0.001), IL-2 (OR = 7.35, 95% CI: 1.316~49.527, *P* = 0.030), and TNF-α (OR = 1.82, 95% CI: 1.262~2.728, *P* = 0.002) were independent risk factors for the occurrence of irAE (**Table 4**).

**Table 4:** Multivariate logistic regression analysis of irAEs occurrence.

Variables	β	S.E	Z	*P*	OR(95%CI)
CRP	1.595	0.285	5.605	<0.001	4.930(2.975-9.184)
IL-4	1.722	0.614	2.806	0.005	5.597(1.757-19.908)
NLR	1.714	0.462	3.714	<0.001	5.55(2.36-14.629)
IL-2	1.995	0.918	2.173	0.030	7.349(1.316-49.527)
TNF-α	0.596	0.195	3.054	0.002	1.815(1.262-2.728)
MLR	2.330	4.233	0.551	0.582	10.278(1.004-11.611)
Combined chemotherapy	0.800	0.597	1.341	0.180	2.225(0.706-7.488)
COPD	0.997	0.706	1.413	0.158	2.710(0.693-11.320)

### Sensitivity analysis

To assess the potential impact of unmeasured confounding factors on the main research results, we calculated the E value of the strongest predictor (IL-2). The observed OR for IL-2 was 7.349 (95% CI: 1.316~49.527), and the E-value of this point estimate was 14.18, while the E-value of the lower confidence interval was 1.96.

### Construction of the nomogram, performance evaluation and calibration

**Figure 4:**
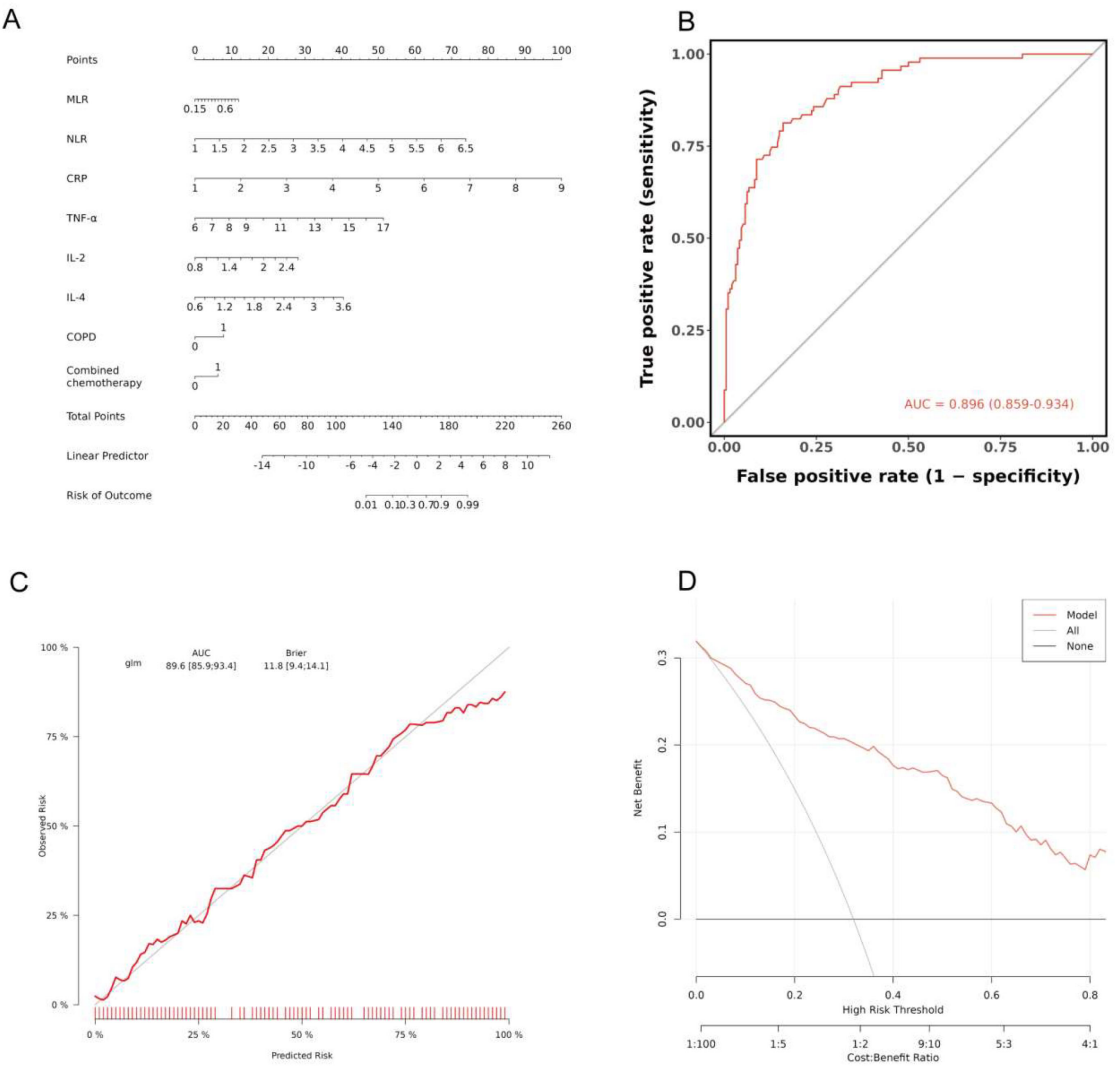
Construction and validation of the nomogram prediction model. **(A)** Line graph; **(B)** ROC curve; **(C)** Calibration curve; **(D)** Decision curve.

CRP, IL-4, NLR, IL-2, TNF-α, MLR, combined chemotherapy and COPD were used to create a nomogram model based on the findings of the multivariate logistic regression analysis. The individual prediction probability (Pr) of irAE was quantified using the nomogram scoring system, as shown in **Figure 4A**.

The subjects’ AUC was 0.896 (95% CI: 0.859~0.934) (**Figure 4B**), suggesting that the prediction model performs well. The internally validated AUC was 0.891 (95% CI 0.851~0.930).

The calibration curve has a high degree of conformity to the ideal diagonal (**Figure 4C**). The Brier score is 11.8% (95% CI: 9.4~14.1). The calibration slope was 0.987 (95% CI: 0.834~1.140) and the calibration intercept was -0.012 (95% CI: −0.089~0.065). The Hosmer-Lemeshow test yielded a non-significant result (*χ²* = 11.80, df=8, *P* = 0.160), suggesting no evidence of poor calibration according to this specific test. The DCA curve in **Figure 4D** shows a net benefit greater than 0 across the entire threshold range of 0.00–0.08. The slope and intercept were estimated through the internal self-re-sampling method; before this model is considered for clinical application, these estimated values must be independently verified in an external validation cohort.

### SHAP interpretability analysis

**Figure 5:**
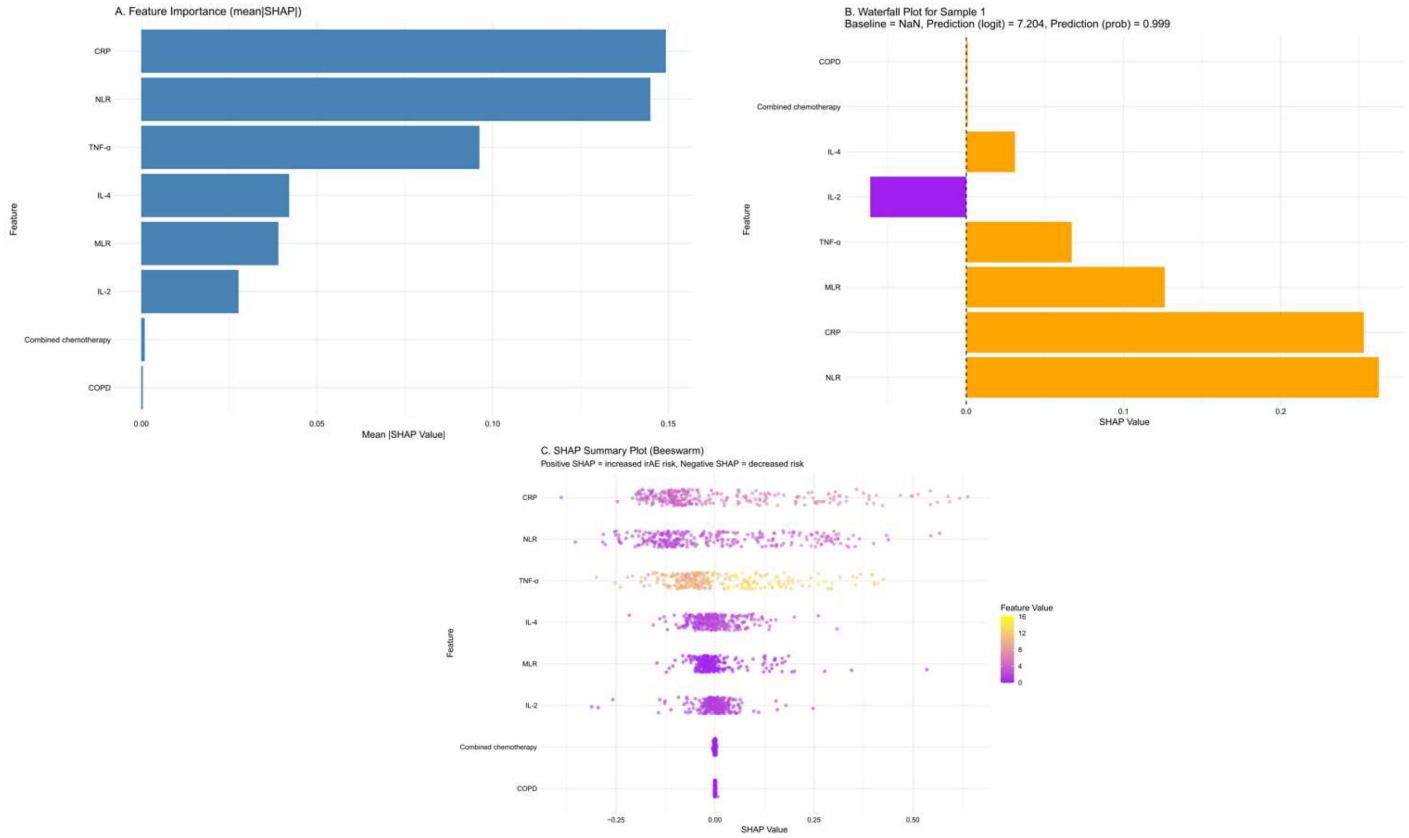
SHAP analysis. **(A)** SHAP feature importance bar plot, quantifying the contribution of each predictor to irAE occurrence using average absolute SHAP values. **(B)** SHAP waterfall plot, illustrating the risk prediction process of irAE for individual patients and visually demonstrating the impact of variables on final predicted values. **(C)** SHAP bee swarm plot, showing the correlation between variable values and SHAP values across all samples, where red indicates high variable values and purple indicates low values. Positive SHAP values suggest increased risk of irAE occurrence, while negative values indicate reduced risk.

The interpretability of the irAE prediction model was evaluated through the use of SHAP (SHapley Additive exPlanations) analysis in this study. The feature importance bar chart (**Figure 5A**) shows that the average absolute SHAP value of CRP was the highest, followed by NLR. The contributions of TNF-α, IL-4, MLR, IL-2, combined chemotherapy and COPD decreased successively.

The single-sample Shap waterfall plot (**Figure 5B**) illustrates the situation for a typical patient: this sample represents an extremely high risk of irAE. NLR, CRP, MLR, TNF-α, IL-2, and IL-4 all show positive SHAP contributions, being the core factors contributing to the elevated risk; the features such as combined chemotherapy and COPD have weak contributions and have limited impact on the prediction of this high risk.

The SHAP bee swarm plot (**Figure 5C**) shows the distribution of each feature’s SHAP value in the entire sample and its correlation with the feature values (color represents the feature value level: red for high values, purple for low values). The SHAP bee swarm plot shows that CRP, NLR, TNF-α, IL-4 and IL-2 are the main characteristics influencing the risk of irAE. High values mostly correspond to positive SHAP values, suggesting that an increase will increase the risk of irAE. The SHAP values of combined chemotherapy and COPD are generally small and have a relatively weak contribution to the model prediction, with limited impact.

## Discussion

ICIs have become the core treatment modality for NSCLC significantly improving patient survival outcomes while also causing a series of irAEs (16). IrAEs can affect multiple organs, including the lungs, gastrointestinal tract, skin, and endocrine system. Although mild cases are manageable, severe ones may be life-threatening and represent a major cause of ICIs discontinuation, impacting both efficacy and safety (17). In this study of 285 patients with advanced NSCLC, the incidence of irAEs was 31.93%, with immune-related pneumonia, diarrhea, and skin reactions being the most common. This finding aligns with the 25%–40% incidence reported in real-world studies (18).

Age, gender, history of COPD, history of autoimmune diseases, type of immunotherapy, combined chemotherapy, CRP, TNF-α, IL-2, IL-4, IL-6, NEU, LYM, MLR, NLR, PLR, SII, and ORR were all statistically significantly different between the irAE group and the non-irAE group (*P* < 0.05). These variables may influence the development and occurrence of irAEs. Patients in the irAE group were generally older than those in the non-irAE group. This could be linked to the deterioration of immune function reserve in older patients, resulting in aberrant regulatory T cell function and raising the possibility of immune overactivation following ICI treatment (19). Regarding gender differences, male patients in this study showed a higher observed incidence of irAEs. Some studies have hypothesized that estrogen may be involved in regulating immune response intensity through effects on antigen presentation and cytokine secretion, which could potentially contribute to this difference, but the underlying mechanisms remain to be confirmed (20). Additionally, patients with combined COPD or autoimmune disease history had a significantly higher incidence of irAEs. Chronic airway inflammation related to COPD may make lung tissue more sensitive to immune attack, while a history of autoimmune disease indicates that the immune tolerance mechanism in the body has defects (21, 22).

In clinical treatment characteristics, the type of immunotherapy and combined chemotherapy were significantly associated with the occurrence of irAEs. Different ICIs (such as PD-1 inhibitors and PD-L1 inhibitors) may have different toxicity spectra due to their target and affinity differences; combined chemotherapy can enhance the anti-tumor effect while also activating the immune system through chemotherapy-induced cell death, further activating the immune system and possibly exacerbating the risk of irAE (23). Before treatment, the irAE group’s levels of inflammatory-related cytokines, including CRP, TNF-α, IL-2, IL-4, and IL-6, were considerably greater than those of the non-irAE group, according to laboratory markers. CRP, as a sensitive marker of systemic inflammation, its increase reflects the body’s pro-inflammatory state, which may indicate that the immune system is more likely to be activated by ICIs (24). TNF-α, IL-2, IL-4,and IL-6 are considered core mediators of the inflammatory cascade (25, 26). Regarding peripheral blood cell counts and derived ratios, the irAE group exhibited significantly higher LYM, MLR, NLR, PLR and SII, and lower NEU, compared with the non-irAE group. These observations are consistent with a profile of heightened innate immune activity and reduced adaptive immune function, though such interpretations remain correlational. Furthermore, high NLR and high SII have been linked to poor prognoses in various solid tumors in prior reports (27).

In this investigation, PFS and OS did not show any statistically significant differences between the irAE and non-irAE groups. The median OS was 285 days in the irAE group and 379 days in the non-irAE group; the median PFS was 242 days in the irAE group and 332 days in the non-irAE group. These results are in line with some other observational studies that found no apparent distinctions in survival between NSCLC patients whether or not they received irAE (28). The landmark method confirmed comparable PFS and OS between the irAE and non-irAE groups before and after the cutoff time points. These findings align with several observational studies that failed to detect survival differences associated with irAEs in NSCLC. This phenomenon may be influenced by the following factors: Considerable heterogeneity was present in the irAE group due to the varying severity and types of irAEs. Severe irAEs, such as immune-related pneumonia, can lead to treatment interruption and a poor prognosis, which could offset the potential survival benefit of milder irAEs (28). Furthermore, we designed this study as an observational study at a single center, and we used a limited sample size. This means that the statistical power may have been insufficient to detect subtle survival differences. Some large cohort studies and meta-analyses have reported an association between irAEs and improved survival following immunotherapy for NSCLC. However, these findings are largely based on mild or specific types of irAEs (29).

When developing the nomogram, we followed predictive modeling principles by prioritizing overall model performance over the statistical significance of individual variables. It is worth noting that independent risk factors are used to explore the etiology, while the nomogram serves as a comprehensive scoring system to improve predictive accuracy. The final model incorporated eight predictors: CRP, IL-4, NLR, IL-2, TNF-α, MLR, combined chemotherapy and COPD. Among these, CRP, IL-4, NLR, IL-2 and TNF-α were identified as independent predictors of irAEs with significant associations, whereas MLR, combined chemotherapy and COPD showed no statistical significance in multivariate logistic regression. MLR has been confirmed in previous studies to be associated with the risk of irAEs. Michailidou et al. (30) conducted a retrospective analysis of 470 patients treated with ICI. In this study, MLR did not reach a significant association with the sample characteristics; combined chemotherapy has been proven to increase the risk of irAEs, and a network meta-analysis showed that ICI combined with chemotherapy significantly increased the risk of any grade and grade 3–5 irAEs compared to single-agent treatment (31). This study did not reach a significant association affected by the proportion of combined chemotherapy in the sample and the specific composition of the scheme; COPD, as a representative of chronic systemic inflammatory state, although there is limited direct evidence in the current literature as an independent predictor of generalized irAEs (32), there is a biological association hypothesis between it and immune checkpoint inhibitor-related pneumonia, retaining this variable is helpful to reflect the patient’s comorbidity burden and baseline inflammatory state.

Compared with previously published irAE prediction models that rely heavily on routine inflammatory ratios such as NLR, PLR, and SII, our model further incorporates multiple circulating cytokines (TNF-α, IL-2, IL-4) and CRP, offering a more comprehensive reflection of the systemic inflammatory and immune activation status before ICI initiation. In terms of discriminative performance, the AUC of 0.896 (95% CI: 0.859–0.934) in the original cohort and 0.891 after bootstrap correction. This improvement may partly result from the inclusion of multiple cytokine markers alongside conventional inflammatory indices, which together capture different aspects of the immune-inflammatory axis involved in irAE pathogenesis. The contribution of non-significant variables (MLR, combined chemotherapy, COPD) to the nomogram’s overall performance was modest in our SHAP analysis, where CRP and NLR exhibited the strongest predictive impact, followed by TNF-α, IL-4, and IL-2. The inclusion of these non-significant factors does not appear to have affected the model’s calibration, as evidenced by the good fit between the calibration curve and the ideal straight line, as well as the non-significant results of the Hosmer-Lemeshow test.

### Limitations

However, this study is a single-center observational study with a limited sample size, which may introduce selection bias; the model has not yet been externally validated, and its generalization ability needs to be verified through multi-center, large-sample prospective cohort studies. Due to the relatively limited sample size and event count, this study employed LASSO regression for variable selection to avoid overfitting. In addition, several clinical variables, including age, sex, type of immune checkpoint inhibitor and history of autoimmune disease, showed significant differences in the univariate analysis, but were excluded from the LASSO regression. These unaccounted factors may introduce residual confounding into the final predictive model. Only the single variable IL-2 was analyzed using the E-value method, which does not validate the robustness of the entire model in the presence of unmeasured confounders. Therefore, residual confounding remains an unavoidable limitation of this study. Since the full modeling pipeline (variable selection + model fitting) was not repeated within each bootstrap sample, the AUC and other performance indicators obtained from bootstrap validation may be slightly optimistic, and the risk of potential overfitting cannot be completely ruled out. Future external validation based on independent cohorts, particularly through larger sample sizes and multi-center designs, is essential to further elucidate the independent roles of these clinical factors in predicting irAEs. Secondly, due to the low incidence of severe adverse events and the relatively small number of examined samples, subgroup analysis could not be conducted. Furthermore, some delayed irAE cases may have been underestimated because the median follow-up period in this study was just 7.81 months. Furthermore, the median follow-up time of 7.81 months in this cohort may have led to an underestimation of delayed-onset irAEs. Therefore, the accuracy of this model in predicting delayed toxicity still needs to be further validated in studies with longer follow-up periods. Fourth, as a retrospective study, continuous measurements of inflammatory biomarkers during treatment were not systematically collected. Consequently, it was not possible to analyze the dynamic changes in biomarkers at the time of imaging assessment or during the early stages of treatment. This limits our ability to assess whether trends in biomarker changes during treatment can further improve the predictive accuracy of irAEs beyond baseline values. Future prospective studies should incorporate planned longitudinal biomarker sampling to address this limitation. Future research should focus on conducting multi-center prospective studies, including continuous dynamic inflammatory and immune monitoring indicators, to build a more accurate prediction model, and explore preventive intervention strategies based on risk stratification to reduce the incidence of irAE in high-risk patients.

## Conclusions

In conclusion, this investigation discovered that CRP, NLR, TNF-α, IL-4 and IL-2 prior to treatment were independent predictors of irAE in NSCLC patients undergoing ICI therapy. The nomogram model built using these indicators shows promising discriminatory power and predictive accuracy in this single−center derivation cohort. Nevertheless, due to the lack of external validation, the model should currently be considered exploratory and hypothesis−generating. Its potential to assist clinicians in risk stratification and monitoring planning needs to be confirmed in independent multicenter cohorts before any clinical implementation.

## Data Availability

The raw data supporting the conclusions of this article will be made available by the authors, without undue reservation.

## References

[1] Kuang Y Zu K Xing D Liu A Uyei J Nevo A . Incidence of pneumonitis in Asian patients with lung cancer: A systematic literature review and meta-analysis. Clin Lung Cancer. (2026) 27:25–37. doi: 10.1016/j.cllc.2025.11.019 41539026

[2] Lu Y Fang D Guo J Huang H . Partial transformation from non-small cell lung cancer to small cell lung cancer: A case report and literatures review. Front Oncol. (2025) 15. doi: 10.3389/fonc.2025.1441182 40134592 PMC11933710

[3] Yin G Liu X Yu X Tan S Liu F . Analysis of icis alone or in combination rechallenged outcomes after progression from first-line icis plus chemotherapy in patients with advanced non-small cell lung cancer. Sci Rep. (2025) 15:30. doi: 10.1038/s41598-024-83947-7 39747923 PMC11696066

[4] Nityashree KL Rachitha P Hanchinmane S Raghavendra VB . Advancing precision medicine: Uncovering biomarkers and strategies to mitigate immune-related adverse events in immune checkpoint inhibitors therapy. Toxicol Rep. (2025) 14:102035. doi: 10.1016/j.toxrep.2025.102035 40458297 PMC12127610

[5] Zhang Q Chen J Tsai N Zhu X Zhao M Meng L . Characterising immune-related adverse events in different types of cancer among Chinese patients receiving immune checkpoint inhibitors. Sci Rep. (2024) 14:30983. doi: 10.1038/s41598-024-82105-3 39730637 PMC11681080

[6] Naud N Addou S Bouzid D Lebbé C Zalcman G Madelaine I . Factors associated with immune-related adverse events in cancer patients treated with immunotherapy visiting the emergency department. Am J Emergency Med. (2026) 99:17–24. doi: 10.1016/j.ajem.2025.09.010 40957139

[7] Yan D Xu J Wang D Xing Q He X Wang D . Plasma proteome-driven liquid biopsy for individualized monitoring and risk stratification of immune-related adverse events in checkpoint immunotherapy. Mol Cell Proteomics. (2026) 25:101488. doi: 10.1016/j.mcpro.2025.101488 41391830 PMC12818211

[8] Jayathilaka B Mian F Cockwill J Franchini F Au-Yeung G M IJ . Analysis of risk factors for immune-related adverse events induced by immune checkpoint inhibitor treatment in cancer: A comprehensive systematic review. Crit Rev Oncol Hematol. (2025) 207:104601. doi: 10.1016/j.critrevonc.2024.104601 39706233

[9] Raynes G Stares M Low S Haron D Sarwar H Abhi D . Immune-related adverse events, biomarkers of systemic inflammation, and survival outcomes in patients receiving pembrolizumab for non-small-cell lung cancer. Cancers (Basel). (2023) 15(23):5502. doi: 10.3390/cancers15235502 38067207 PMC10705211

[10] Hamakawa Y Hirahara A Hayashi A Ito K Shinohara H Shiba A . Prognostic value of systemic immune-inflammation index in patients with small-cell lung cancer treated with immune checkpoint inhibitors. BMC Cancer. (2025) 25:17. doi: 10.1186/s12885-025-13440-5 39762819 PMC11706134

[11] Yao B Xia G Zhao L Ma K Chen Z . Sii and lung cancer in middle-aged and elderly population: The nonlinear connection and the mediating role of absi. World J Surg Oncol. (2025) 23:345. doi: 10.1186/s12957-025-04005-8 41013636 PMC12465597

[12] Moloney C Demir T Chung LIY Oh Y Chae YK . Comparing rates of immune checkpoint inhibitor toxicity in different treatment settings (neoadjuvant, adjuvant, and palliative) in non-small cell lung cancer (nsclc): A meta-analysis of phase iii clinical trials. J Clin Oncol. (2024) 42:8645. doi: 10.1200/JCO.2024.42.16_suppl.8645 42148471

[13] Chakrabarty N Mahajan A Shetty N Mummudi N Niyogi D Hota F . Imaging patterns and recommendations for diagnosis, staging, and management of lung cancer. BJR Open. (2025) 7:tzaf013. doi: 10.1093/bjro/tzaf013 40486071 PMC12145177

[14] Schneider BJ Naidoo J Santomasso BD Lacchetti C Adkins S Anadkat M . Management of immune-related adverse events in patients treated with immune checkpoint inhibitor therapy: Asco guideline update. J Clin Oncol. (2021) 39:4073–126. doi: 10.1200/jco.21.01440 34724392

[15] Freites-Martinez A Santana N Arias-Santiago S Viera A . Using the common terminology criteria for adverse events (ctcae - version 5.0) to evaluate the severity of adverse events of anticancer therapies. Actas Dermosifiliogr (Engl Ed). (2021) 112:90–2. doi: 10.1016/j.ad.2019.05.009 32891586

[16] Giommoni E Pillozzi S Ottanelli C Cosso F Caliman E Mazzoni F . Eosinophil count and immune-related adverse events (iraes) as predictive biomarkers of survival in patients with nsclc treated with icis. J Clin Oncol. (2023) 41:e21144. doi: 10.1200/JCO.2023.41.16_suppl.e21144 42148471

[17] Smith MR Wang Y Dixon CB D'Agostino R Liu Y Ruiz J . Mutations associated with high-grade iraes in nsclc patients receiving immunotherapies. Clin Lung Cancer. (2024) 25:e379–e88. doi: 10.1016/j.cllc.2024.07.003 39095235 PMC11588554

[18] Pasello G Pavan A De Nuzzo M Frega S Ferro A Dal Maso A . Immune-related adverse events in patients treated with immunotherapy for locally advanced or metastatic nsclc in real-world settings: A systematic review and meta-analysis. Front Oncol. (2024) 14:1415470. doi: 10.3389/fonc.2024.1415470 39045561 PMC11263096

[19] Gao J Yuan Y Wang X He L Li L . Impact of immunosenescence on immune-related adverse events in elderly patients with cancer. Aging Med (Milton). (2025) 8:e70000. doi: 10.1002/agm2.70000 40012861 PMC11864504

[20] Duma N Abdel-Ghani A Yadav S Hoversten KP Reed CT Sitek AN . Sex differences in tolerability to anti-programmed cell death protein 1 therapy in patients with metastatic melanoma and non-small cell lung cancer: Are we all equal? Oncologist. (2019) 24:e1148–e55. doi: 10.1634/theoncologist.2019-0094 31036771 PMC6853107

[21] Li F Zheng L Xu X Jin J Li X Zhou L . The impact of chronic obstructive pulmonary disease on the risk of immune-related pneumonitis in lung cancer patients undergoing immunotherapy: A systematic review and meta-analysis. BMC Pulm Med. (2024) 24:393. doi: 10.1186/s12890-024-03180-w 39143553 PMC11323643

[22] de Torres CS Trallero-Araguas E Marmolejo D Hermida JMU Mejía ND Baselga PM . Balancing risks and benefits of immune checkpoint inhibitors in patients with pre-existing immune-mediated inflammatory diseases: A comprehensive review. Crit Rev Oncol Hematol. (2025) 216:105000. doi: 10.1016/j.critrevonc.2025.105000 41176078

[23] Heyward J Lesko CR Murray JC Mehta HB Segal JB . Harm-benefit balance of immune checkpoint inhibitors in non-small cell lung cancer. JAMA Oncol. (2025) 11:707–16. doi: 10.1001/jamaoncol.2025.0985 40338588 PMC12062994

[24] Duvall LE . The use of peripheral blood biomarkers for predicting the risk of immune-related adverse events in immune checkpoint inhibitor therapy. Ann Clin Biochem. (2025) 62:236–56. doi: 10.1177/00045632241312629 39715717

[25] Husain B Kirchberger MC Erdmann M Schüpferling S Abolhassani AR Fröhlich W . Inflammatory markers in autoimmunity induced by checkpoint inhibitors. J Cancer Res Clin Oncol. (2021) 147:1623–30. doi: 10.1007/s00432-021-03550-5 33837821 PMC8076116

[26] Lacouture ME Goleva E Shah N Rotemberg V Kraehenbuehl L Ketosugbo KF . Immunologic profiling of immune-related cutaneous adverse events with checkpoint inhibitors reveals polarized actionable pathways. Clin Cancer Res. (2024) 30:2822–34. doi: 10.1158/1078-0432.Ccr-23-3431 38652814 PMC11215405

[27] Ma G Ma L Zhang Y Chen Y Zhang Y Guo W . Prognostic significance of the lymphocyte-to-high-density lipoprotein ratio in long-term efficacy of combined immunotherapy for advanced non-small cell lung cancer. Front Oncol. (2025) 15:1712779. doi: 10.3389/fonc.2025.1712779 41347091 PMC12672221

[28] Nojiri T Omura A Nishimura H Iede K Takeyasu Y Katsushima U . Severe immune-related adverse events and their effect on survival in patients with advanced non-small cell lung cancer receiving immune checkpoint inhibitors. Cancer Diagn Progn. (2026) 6:106–13. doi: 10.21873/cdp.10511 41487923 PMC12758705

[29] Cook S Samuel V Meyers DE Stukalin I Litt I Sangha R . Immune-related adverse events and survival among patients with metastatic nsclc treated with immune checkpoint inhibitors. JAMA Netw Open. (2024) 7:e2352302. doi: 10.1001/jamanetworkopen.2023.52302 38236598 PMC10797458

[30] Michailidou D Khaki AR Morelli MP Diamantopoulos L Singh N Grivas P . Association of blood biomarkers and autoimmunity with immune related adverse events in patients with cancer treated with immune checkpoint inhibitors. Sci Rep. (2021) 11:9029. doi: 10.1038/s41598-021-88307-3 33907229 PMC8079370

[31] Yin Q Wu L Han L Zheng X Tong R Li L . Immune-related adverse events of immune checkpoint inhibitors: A review. Front Immunol. (2023) 14:1167975. doi: 10.3389/fimmu.2023.1167975 37304306 PMC10247998

[32] Riondino S Rosenfeld R Formica V Morelli C Parisi G Torino F . Effectiveness of immunotherapy in non-small cell lung cancer patients with a diagnosis of copd: Is this a hidden prognosticator for survival and a risk factor for immune-related adverse events? Cancers (Basel). (2024) 16(7):1251. doi: 10.3390/cancers16071251 38610929 PMC11011072

